# Clinical response to chemoradiotherapy in esophageal carcinoma is associated with survival and benefit of consolidation chemotherapy

**DOI:** 10.1002/cam4.3273

**Published:** 2020-07-06

**Authors:** Zongxing Zhao, Yanan Zhang, Xin Wang, Xiaotao Geng, Liqiong Zhu, Minghuan Li

**Affiliations:** ^1^ School of Medicine Shandong University Jinan China; ^2^ Department of Radiation Oncology Liaocheng People’s Hospital Liaocheng China; ^3^ Department of Radiation Oncology Shandong Cancer Hospital and Institute Jinan China; ^4^ Department of Health Care Liaocheng People’s Hospital Liaocheng China; ^5^ Shandong First Medical University and Shandong Academy of Medical Sciences Jinan China

**Keywords:** chemoradiotherapy, clinical response, consolidation chemotherapy, esophageal carcinoma, prognosis

## Abstract

**Purpose:**

Few studies have reported the impact of the clinical response of patients with Esophageal Carcinoma to chemoradiotherapy (CRT). Our study examines the association between clinical response and pretreatment variables, survival, patterns of failure, and benefit of consolidation chemotherapy in subjects with esophageal squamous cell carcinoma (ESCC) patients receiving CRT.

**Methods:**

Data from ESCC patients treated at Shandong Cancer Hospital between January 2013 and December 2016 were analyzed retrospectively. By definition, we considered a poor response as progressive disease (PD) and stable disease (SD), while complete response (CR) and partial response (PR) were considered as a good response. Multivariate analyses were carried out using Cox proportional hazards models and patient survival was assessed using the Kaplan‐Meier and log‐rank test.

**Results:**

After CRT, 136 (48.9%) patients responded well (good response) and 152 (51.1%) patients responded poorly (poor response). Overall survival (OS) and progression‐free survival (PFS) differed significantly between patients responded well and those responded poorly. Patients with an early‐stage or the upper location of the tumor were more likely to achieve a good response. Patients showing poor responses tended to experience local failure. The 3‐year OS and PFS rates of patients showing poor response were 38.9% and 25.5%, respectively, for the CRT with consolidation chemotherapy (CRT + C) group, and 22.7% and 16.7%, respectively, for the CRT group. However, patients with a good response did not benefit from the consolidation chemotherapy. Primary tumor location, T category, N category, and clinical response to chemoradiotherapy were independent factors predicting OS and PFS in ESCC.

**Conclusion:**

Clinical response to CRT substantially improves patient survival and is associated with failure patterns in ESCC. Consolidated chemotherapy may benefit patients with a poor response.

## Graphical abstract

Our research analyzed the association of clinical response with pretreatment variables, survival, patterns of failure, and benefit of consolidation chemotherapy in esophageal squamous cell carcinoma (ESCC) patients treated with CRT. We found that clinical response to CRT is associated with significantly survival and failure patterns in ESCC patients. Consolidated chemotherapy may benefit patients with a poor response.

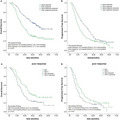

## 1 INTRODUCTION

Globally, esophageal cancer (EC) was ranked seventh in terms of incidence and sixth in terms of mortality in 2018. In Asian, Esophageal squamous cell carcinoma (ESCC) is the most frequently diagnosed EC.^1^ ESCC in which patients are not eligible for surgery is often treated with a combination of concurrent chemotherapy plus radiotherapy. Currently, chemoradiotherapy (CRT) only results in a 5‐year survival rate of 10%‐30%.^2^, ^3^, ^4^, ^5^ However, it remains unclear how locally advanced ESCC patients respond to consolidation chemotherapy plus CRT. Since the RTOG 94‐05 trial,^5^ combining four courses of chemotherapy with radiotherapy has been recommended as the standard treatment in ESMO Guidelines.^6^ However, Chen et al revealed that consolidation chemotherapy did not improve overall survival (OS) after CRT.^7^

Complete pathologic response (pCR) to neoadjuvant CRT results in prolonged survival compared with patients showing incomplete pathological response.^8^, ^9^, ^10^ The impact of clinical outcomes in patients receiving chemoradiotherapy in nonsurgical esophageal carcinoma is unclear. Herein, we retrospectively reviewed our experience with a clinical response associated with pretreatment clinical variables, patterns of failure, and survival outcomes.

## 2 MATERIAL AND METHODS

### 2.1 Patients

We retrospectively reviewed 278 patients with histologically confirmed ESCC who underwent CRT at Shandong Cancer Hospital between January 2013 and December 2016. Patients with T2‐4N0‐3 disease were eligible. Data including age, gender, tumor location, and staging were collected. Pretreatment evaluations included barium swallow, enhanced computed tomography (CT), esophageal ultrasound, and/or positron emission tomography, when feasible. The American Joint Committee on Cancer system (8th edition) for esophageal carcinoma was employed to perform clinical staging. The Medical Ethics Committee of Shandong Cancer Hospital approved this study.

### 2.2 Treatment

All patients of this study were given concurrent platinum‐based chemotherapy of paclitaxel or 5‐fluorouracil. The concurrent chemotherapy regimen consisted of cisplatin (75 mg/m^2^; intravenously infusion on day 1) in combination with 5‐fluorouracil (750 mg/m^2^; intravenous infusion daily for 24 h on days 1‐4) or paclitaxel (135 mg/m^2^; intravenously infusion on day 1) every 4 weeks. The doses of the consolidation chemotherapy were the same as those in the concurrent chemotherapy regimen. The clinical target volumes were defined as the cranial, caudal margin of 3 cm of a primary tumor, the lateral, anterior, and posterior borders of 0.8 cm of a primary tumor, and regional lymph node area at risk of microscopic disease. Patients received a dose of 50.4‐66Gy using intensity‐modulated radiation therapy. Consolidation chemotherapy was conducted 4 weeks after the completion of CRT Patients were divided into two groups based on the chemotherapy cycles: the CRT plus consolidation chemotherapy (CRT + C) group comprised patients who were given four courses of chemotherapy combined with radiation therapy, and the CRT group was made up of individuals who received less than four courses of chemotherapy. Eight patients experienced progressive disease (PD) in the course of treatment, and their treatment was replaced with second‐line chemotherapy.

### 2.3 Evaluation and surveillance

Patients received follow‐up physical examination, such as barium swallow, endoscopy, and enhanced CT, beginning 1 month after the completion of CRT, at every 3 months in the first 2 years, and every 6 months after that until death or loss of follow‐up. Response Evaluation Criteria in Solid Tumors (RECIST 1.0) system was employed to assess the clinical response and classified as PD, complete response (CR), partial response (PR), and stable disease (SD). A good response was defined as a CR plus a PR and a poor response as SD plus PD. Failure within the radiation treatment volume that encompassed prophylactic nodal coverage was considered local, and failure outside the radiation treatment volume was regarded as distant. OS was considered as the period from initiation of therapy to the date of last follow‐up or mortality. Distant metastasis‐free survival (DMFS) was considered as the time between treatment initiation and disease progression. Locoregional relapse‐free survival (LRRFS) was also assessed.

### 2.4 Statistical analysis

Data analysis was conducted using SPSS 23.0 (SPSS, Chicago, IL, USA). We employed Fisher's exact test to compare distributions of pretreatment variables between responders and nonresponders. The associations between patient survival and clinical parameters were assessed using univariate and multivariate Cox proportional hazards models. *P*‐values ≤ 0.05 were considered statistically significant.

## 3 RESULTS

### 3.1 Patient characteristics

The 278 study participants had a median age of 61 years (range, 21‐84 years). Two hundred and thirty (83%) patients were male, and 48 (17%) were female. For treatment, 87 patients received CRT and 191 received CRT plus consolidation chemotherapy. The majority of the ECs occurred in the upper third of the esophagus (49%) and had a T3 primary (60%). Table 1 summarizes the patient characteristics.

**TABLE 1:** Patient and tumor characteristics

Variables	Total no. of cases (% of total)	No. of PR + CR cases (%)	No. of SD + PD cases (%)	*P* value
Age (years)				
＜60	103 (37.1)	53 (51.5)	50 (48.5)	.513
≥60	175 (62.9)	83 (47.4)	92 (52.6)	
Gender				
Male	230 (82.7)	111 (48.3)	119 (51.7)	.630
Female	48 (17.3)	25 (52.1)	23 (47.9)	
Primary tumor location				
Upper	135 (48.6)	79 (58.5)	56 (41.5)	.002
Middle	102 (36.7)	45 (44.1)	57 (55.9)	
Lower	41 (14.7)	12 (29.3)	29 (70.7)	
T category				
T2	52 (18.7)	32 (61.5)	20 (38.5)	.022
T3	167 (60.1)	83 (49.7)	84 (50.3)	
T4	59 (21.2)	21 (35.6)	38 (64.4)	
N category				
N0	60 (21.6)	35 (58.3)	25 (41.7)	.044
N1	121 (43.5)	60 (49.6)	61 (50.4)	
N2	81 (29.1)	38 (46.9)	43 (53.1)	
N3	16 (5.8)	3 (18.8)	13 (81.2)	
Tumor length				
<5 cm	160 (57.6)	81 (50.6)	79 (49.4)	.508
≥5 cm	118 (42.4)	55 (46.6)	63 (53.4)	

Note

PR, partial response; CR, complete response; SD, stable disease; PD, progressive disease.

### 3.2 Response and failure patterns

After CRT, 136 (48.9%) patients responded well (good response), and 142 (51.1%) patients responded poorly (poor response). Fisher's exact test revealed that patients in the good response group tended to have early‐stage ESCC. Patients with tumors at the upper location were more likely to achieve a good response. Notably, there were no significant differences in age and tumor length between the two groups (Table 1). Additionally, 125 patients (45.0%) exhibited distant failure, 158(56.8%) developed local failure, and 51 (18.3%) had no evidence of disease. Among the patients with local failure, 64 responded well and 94 responded poorly. The rate of local recurrence differed significantly between the two groups. Sixty‐six patients (48.5%) who responded well and 59 patients (42.5%) who responded poorly had distant metastases (Table 2).

**TABLE 2:** Correlation between response and patterns of failure

Patterns of failure	Good response	Poor response	*P*
Local failure			
Yes	64/136 (47.1%)	94/142 (66.2%)	.001
No	72/136 (52.9%)	48/142 (33.8%)	
Distant failure			
Yes	66/136 (48.5%)	59/142 (41.5%)	.242
No	70/136 (51.5%)	83/142 (58.5%)	

### 3.3 Survival

**FIGURE 1:**
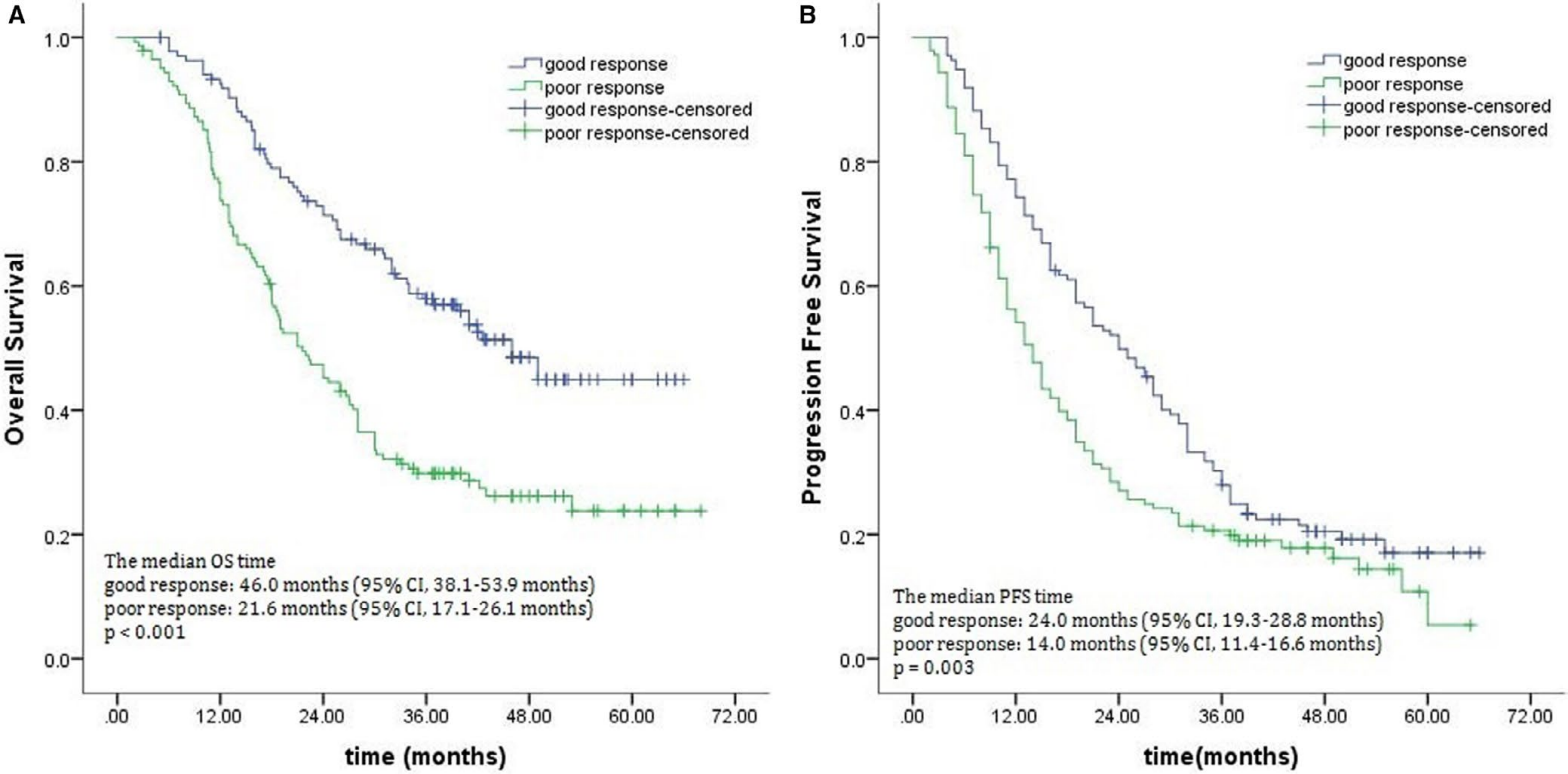
Overall survival (A) and progression‐free survival (B) in patients showing good and poor clinical response to chemoradiotherapy, as determined by Kaplan‐Meier survival analyses

Within the entire cohort, the respective OS and progression‐free survival (PFS) at 1, 3, and 5 years were 82.9 and 64.0%, 43.1 and 24.2%, and 33.6 and 12.0%, respectively. Of the 278 patients, 167 had died by the last follow‐up, including 102 responded poorly and 65 responded well. We found that OS (*P* < .001) and PFS (*P* < .001) differed significantly between the two groups. The 3‐year OS rates were 57.1% and 28.7% for good responders and poor responders, respectively, while their median survival times were 46.0 and 21.6 months, respectively (Figure 1A). The 3‐year PFS rates were 28.0% for good responders and 20.6% for poor responders (Figure 1B). Survival rates based on treatment response are shown in Figure 1.

**FIGURE 2:**
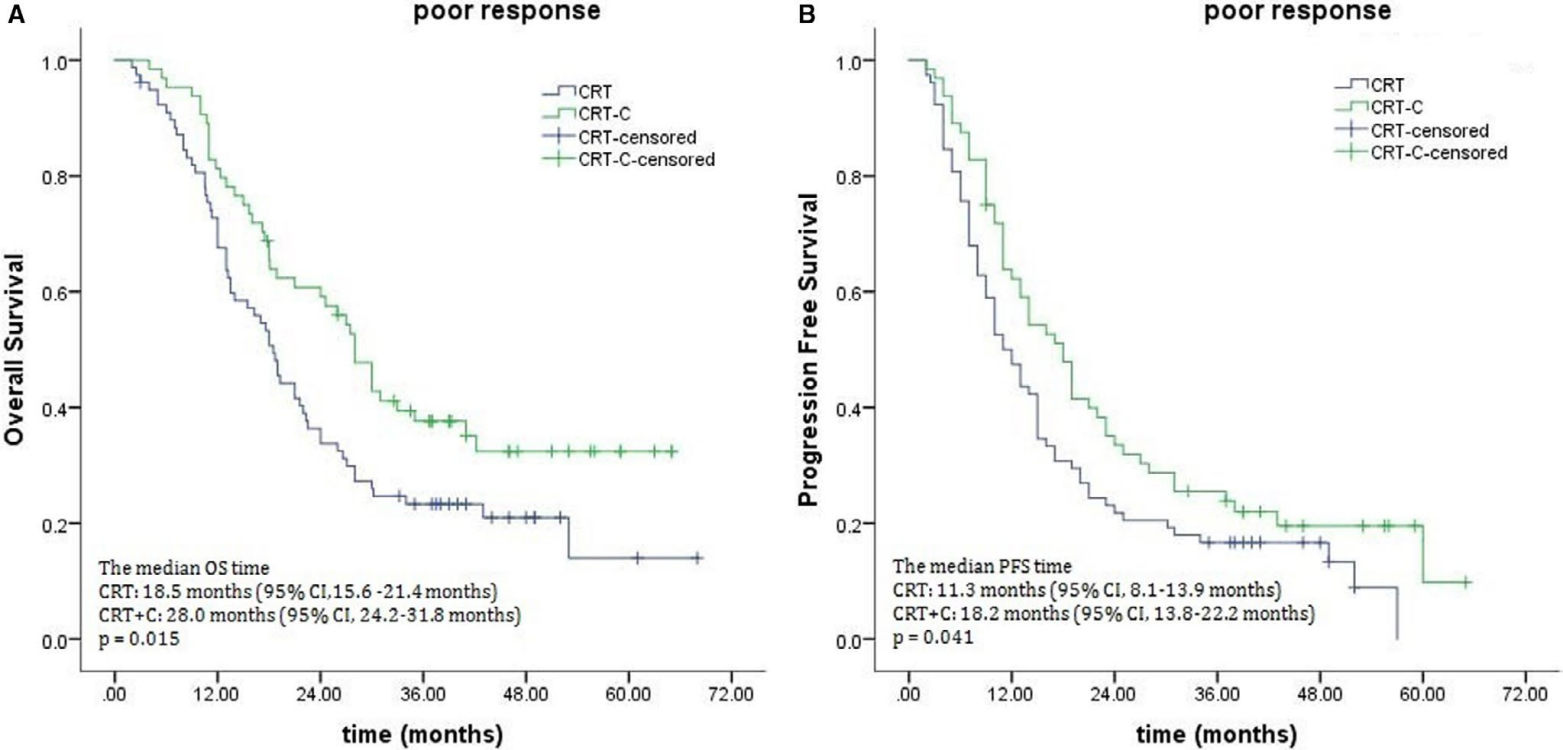
Effects of consolidation chemotherapy on Overall survival (A) and progression‐free survival (B) for patients showing a poor response to chemoradiotherapy

**FIGURE 3:**
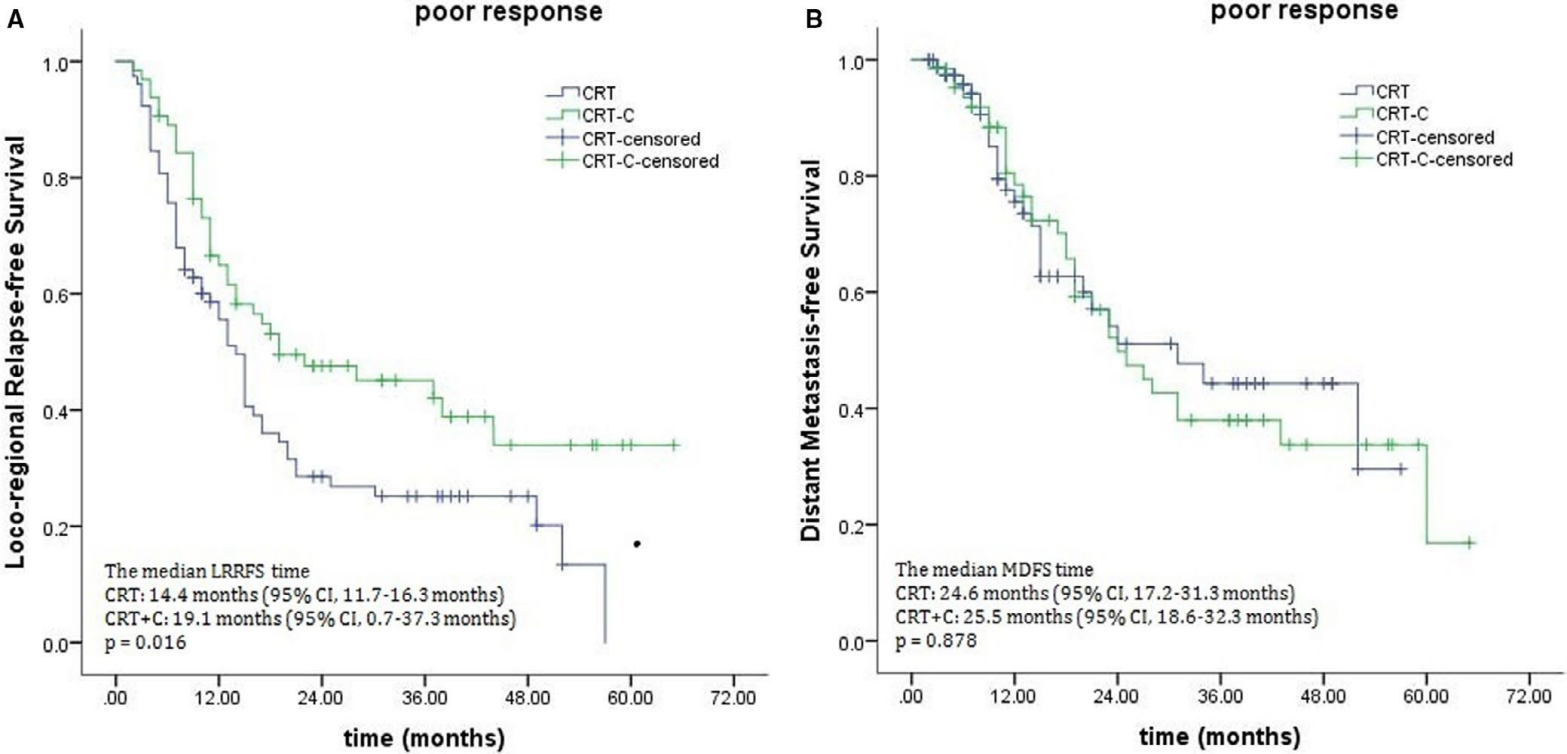
Effects of consolidation chemotherapy on locoregional relapse‐free survival (A) and distant metastasis‐free survival (B) for patients showing a poor response to chemoradiotherapy

**FIGURE 4:**
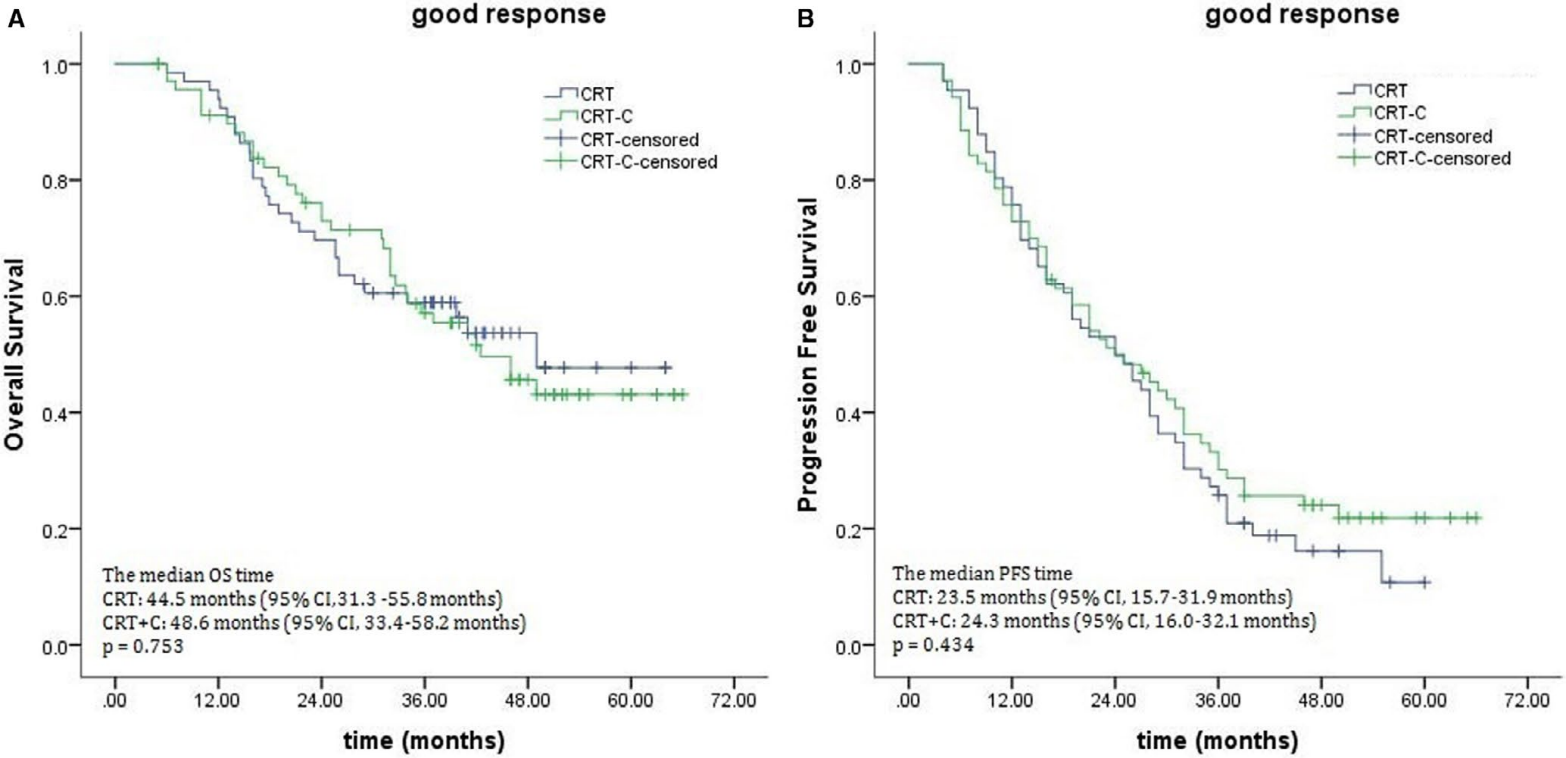
Effects of consolidation chemotherapy on overall survival (A) and progression‐free survival (B) for patients showing a good response to chemoradiotherapy

When the potential benefit of consolidation chemotherapy was analyzed based on clinical response to definitive CRT, marked differences were noted between the treatment outcomes in the poor response subgroup. The 3‐year OS rates were 38.9% for the CRT‐C group and 22.7% for the CRT group, with median survival times of 28.0 and 18.5 months (*P* = .015; Figure 2A). The 3‐year PFS rates for the CRT‐C and CRT groups were 25.5% and 16.7%, respectively, and their corresponding median PFS times were 18.2 months and 11.3 months (*P* = .041; Figure 2B). There were also marked differences in LRRFS (median times, 19.1 months for the CRT‐C group vs 14.4 months for the CRT group, *P* = .016; Figure 3A). No significant difference was observed in DMFS (median times, 25.5 months for the CRT‐C group vs 24.6 months for the CRT group, *P* = .878; Figure 3B). Unlike patients who showed a poor response, those showing good response to CRT did not benefit from consolidation chemotherapy, 3‐year OS rates: 58.8% for the CRT‐C group vs 54.7% for the CRT group, with corresponding median OS: 48.6 vs 44.5 months (*P* = .753; Figure 4A). The median PFS times were 24.3 months and 23.5 months for the CRT‐C and CRT groups, respectively. The 3‐year PFS rates for both groups were 30.2% and 25.8%, respectively (*P* = .434; Figure 4B).

The results of multivariate analyses for OS and PFS are shown in Table 3. The features included in the Cox proportional hazards model were age, gender, primary tumor location, T category, N category, and clinical response to chemoradiotherapy. Primary tumor location, T category, N category, and clinical response to chemoradiotherapy were independent prognostic factors. The remaining factors had no significant effect on any of the two endpoints.

**TABLE 3:** Summary of multivariate analysis for os and pfs in patients with ESCC

Prognostic factor	OS		*P*	PFS		*P*
	HR	95% CI		HR	95% CI	
Age(＜60 vs ≥ 60)	0.994	0.712‐1.387	.970	0.900	0.679‐1.193	.464
Gender (Male vs Female)	0.945	0.606‐1.474	.805	1.084	0.754‐1.560	.663
Tumor location (baseline, Upper)			.012			.003
Middle	1.244	0.868‐1.785	.235	1.453	1.073‐1.966	.016
Lower	1.936	1.251‐2.997	.003	1.891	1.266‐2.824	.002
T category (baseline, T2)			.011			.004
T3	1.370	0.841‐2.231	.206	1.420	0.960‐2.101	.079
T4	2.160	1.246‐3.744	.006	2.115	1.338‐3.342	.001
N category (N0, N1 vs N2, N3)	1.692	1.219‐2.348	.002	1.477	1.108‐1.970	.008
Clinical response (good vs poor)	1.806	1.305‐2.500	.000	1.387	1.078‐1.697	.043

Note

OS, overall survival; ESCC, esophageal squamous cell carcinoma; HR, hazards ratio; CI, confidence interval; PFS, progression‐free survival.

## 4 DISCUSSION

Although many treatments have been developed for unresectable EC, clinical outcomes of patients with this disease are poor.^2^, ^3^, ^4^, ^5^ The impact of clinical response to chemoradiotherapy is unclear. In our study, the response rate was 48.9% for ESCC patients treated with CRT. The 3‐year OS rate was 57.1% for patients who responded well and 28.7% for those who responded poorly. Patients with a good response had favorable survival outcomes, consistent with previous published reports.^11^ However, only a minority of the patients had good survival. The heterogeneity of prognosis poses a significant challenge to personalized treatment for these patients.

The clinical outcomes of cancer patients are highly dependent on the initial stages of cancer. In conformity with previous studies, the presence of higher clinical T stage and N stage correlated strongly with a lower pathological CR rate and worse OS for patients treated with neoadjuvant CRT.^12^, ^13^ In Blum Murphy et al’s review of 911 EC patients received trimodality therapy at the Texas MD Anderson Cancer Center, patients with higher stages had lower pathological CR rates. Additionally, they found that pCR was associated with improved OS and PFS.^12^ However, few studies have reported the association of clinical response with pretreatment features and survival in ESCC after definitive CRT. Besides the T stage and N stage, we found an association between primary tumor locations and clinical response. Chen Y et al revealed that the of OS of patients with primary tumors located in the middle/lower thoracic esophagus is poorer than those with cervical/upper thoracic disease when both groups were treated with CRT,^7^ possibly due to the lower the location of the tumor, the worse the clinical response to definitive CRT. However, we did not find tumor length to be associated with clinical response.

In our study, 158 patients (56.8%) experienced local failure, 125 (45.0%) had a distant failure, and 51 patients (18.3%) had no evidence of disease. The rate of local recurrence was markedly higher in patients showing poor response. Local control is of high clinical significance, leading to improved clinical survival outcomes ^14^; hence, local consolidative therapy should be considered. Some studies have claimed that high‐dose radiotherapy may improve local control and OS in ESCC patients with a high local recurrence rate.^15^, ^16^, ^17^, ^18^ Chang et al analyzed data from 2061 ESCC patients without distant metastasis who received CRT and found that patients in the high‐dose group (>60 Gy) had a higher 2‐year OS rate (35.47%% vs 26.74%) than those in the low‐dose group (≤60 Gy),^15^ indicating that aggressive therapies may improve survival in patients with poor clinical response. Beyond the tumor stage, varying radiosensitivity was a major cause of a poor response. Future studies are required to select the poor clinical response subgroup and give individualized radiotherapy to improve local control and survival without increasing complications.

Administering consolidation chemotherapy to EC patients after CRT is controversial.^6^, ^7^ According to the ESMO Clinical Practice Guidelines, the combining four cycles of cisplatin /5‐fluorouracil with radiotherapy is the standard CRT regimen.^6^ However, Chen Y et al reviewed 812 patients who underwent CRT and found similar PFS (22.1 vs 22.0 months, *P* = .917) and OS (33.8 vs 31.3 months, *P* = .591) between patients in the observation group and those in consolidation group.^7^ Interestingly, Kim et al ^19^ showed that although adjuvant chemotherapy improved the survival of patients with gross residual disease, it did not affect survival in patients with pCR or those with microscopic residual disease after trimodality therapy. We examined the potential benefit of consolidation chemotherapy based on clinical response to definitive CRT and found that the 3‐year OS rates were 38.9% for the consolidation group and 22.7% for the nonconsolidation group in patients with a poor response. Furthermore, patients in the CRT‐C group had a considerably higher LRRFS rate compared with those in the CRT group. In contrast, patients who responded well did not exhibit any benefit. Our results suggest that chemotherapy could improve LRRFS and OS in patients with a poor response.

Distant recurrence was the most predominant failure pattern for patients with a good response after CRT.^20^ In our study, we found no benefit of consolidation chemotherapy to these patients. Consistent with our findings, a previous study showed that patients with stage III non–small cell lung cancer did not benefit from consolidation chemotherapy after CRT.^21^ In contrast, according to the findings of the PACIFIC trial, durvalumab significantly prolonged the 2‐year OS compared with placebo (66.3% vs 55.6%).^22^ The efficacy of immunotherapy combined with cytotoxic agents for ESCC is currently under clinical investigation.^23^, ^24^, ^25^ These results suggest that immunotherapy has emerged as a promising therapeutic regimen as the second‐line treatment of patients with PD‐L1‐positive (CPS ≥ 10) EC. Currently, numerous ongoing clinical trials (ClinicalTrials.gov; Identifier code: NCT02844075, NCT03278626, and NCT03437200) are assessing the effect of RT combined with immune checkpoint inhibitors in patients with locally advanced EC. The advent of immune checkpoint inhibitors could provide alternative therapeutic options for patients resistant to conventional anticancer therapies.

Several drawbacks concerning this study should be highlighted. First, given the retrospective nature, some information, such as histologic differentiation, was not available in all cases. This missing information may have hampered the prognostic performance of some baseline features. Second, this study was conducted at a single center with a relatively small number of patients might not be very reliable. Finally, our patients did not receive homogenous radiation doses, although the optimal radiotherapy dose for ESCC patients undergoing CRT is not clear.

## 5 CONCLUSIONS

We identified clinical response to CRT as a significant prognostic factor in ESCC patients. Our results showed that patients who responded poorly to CRT had a survival benefit from consolidation chemotherapy. Future large cohort prospective clinical trials are warranted to assess the efficacy of consolidation chemotherapy in ESCC patients receiving CRT.

## DISCLOSURE

The authors have no conflict of interest.

## AUTHORS’ CONTRIBUTIONS

Zongxing Zhao and Minghuan Li analyzed the data and drafted the manuscript. Yanan Zhang, Xin Wang, Xiaotao Geng, and Liqiong Zhu participated in data collection. All authors approved it for publication. All authors read and approved the final manuscript.

## Data Availability

The datasets used and/or analyzed during the current study are available from the corresponding author on reasonable request.
